# Traumatisme isolé du pancréas : à propos de 5 cas

**DOI:** 10.4314/pamj.v4i1.53598

**Published:** 2010-03-15

**Authors:** Hicham El Bouhaddouti, Abdelmalek Ousadden, Karim Ibn Majdoub Hassani, Amal Ankouz, Abdesslam Bouassria, Abdellatif Louchi, Khalid Mazaz, Khalid Ait Taleb

**Affiliations:** 1 Service de chirurgie viscérale, A CHU Hassan II, Fès, Maroc;; 2 Service de chirurgie viscérale, B CHU Hassan II, Fès, Maroc

**Keywords:** Traumatisme, trauma, pancréas, pancreas, prothèse, chirurgie, surgery

## Abstract

Les traumatismes isolés du pancréas sont rares, ils sont présents dans 0,2 à 3 % des traumatismes abdominaux. Leur symptomatologie clinique est atypique. La tomodensitométrie abdominale associée à la pancréatographie par résonnance magnétique permet de faire le diagnostic de la lésion pancréatique et de la rupture du canal pancréatique principal. Le traitement peut être uniquement médical, mais surtout chirurgical et endoscopique lors des atteintes canalaires.

## Introduction

Les traumatismes du pancréas sont rares et fréquemment associés à d’autres lésions dans le cadre d’un traumatisme majeur. Ils constituent 0.2 à 6 % de l’ensemble des traumatismes de l’abdomen [1–3]. Le traumatisme isolé du pancréas (TIP) est peu fréquent et de diagnostic souvent difficile. Certains sont méconnus et ne sont découverts que tardivement au stade de complications [4]. Le traitement des traumatismes pancréatiques dépend essentiellement de l’intégrité ou non du canal de Wirsung [4, 5]. Nous rapportons 5 observations de patients qui ont soufferts de traumatismes isolés du pancréas.

## Patients et observations

### Cas numéro 1

**Figure 1:**
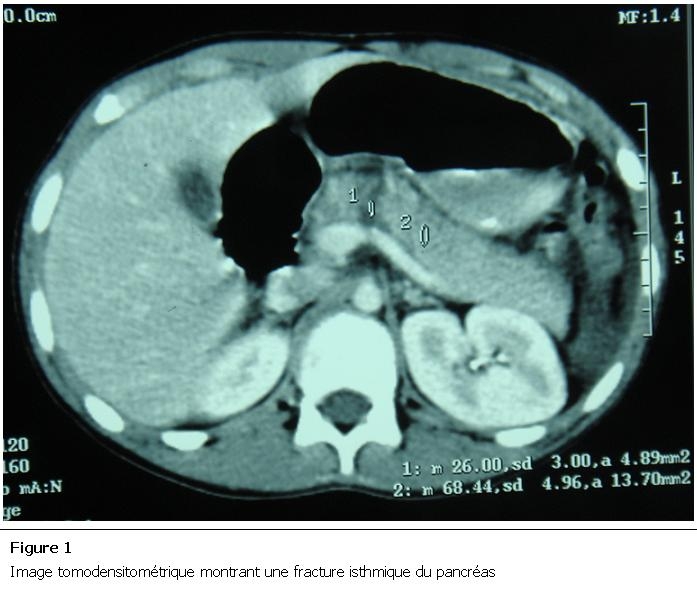
Image tomodensitométrique montrant une fracture isthmique du pancréas

Un cycliste âgé de 17 ans a été victime d’un accident de vélo avec réception du guidon au niveau de l’épigastre occasionnant des épigastralgies. A l’admission, l’abdomen était sensible, la lipasémie était à 10 fois la normale. La TDM montrait une fracture isthmique du pancréas avec une pancréatite stade E de Balthazar [6] (figure 1), le Wirsung traversant la zone d’hyperdensité, sa rupture ne pouvait être écartée (Classe III Lucas [7]). L’attitude était le traitement médical de la pancréatite, avec surveillance clinique et échographique. L’évolution était favorable sans séquelles.

### Cas numéro 2

**Figure 2:**
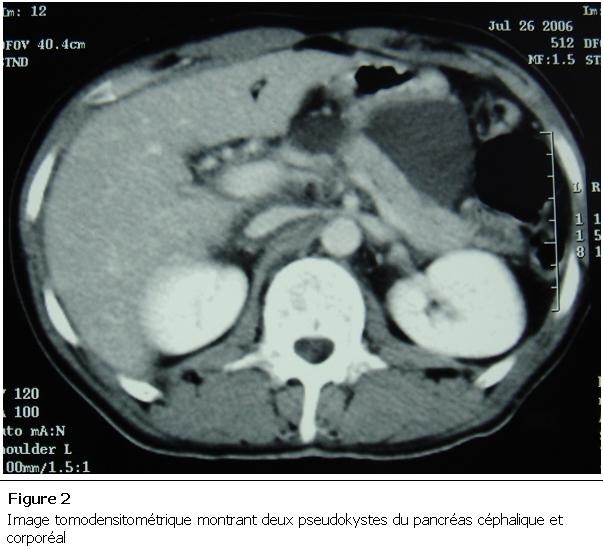
Image tomodensitométrique montrant deux pseudokystes du pancréas céphalique et corporéal

Un homme âgé de 21 ans a été victime d’une agression avec plaie par coup de couteau au niveau de l’hypochondre gauche ayant occasionné chez lui des épigastralgies intermittentes. Le patient n’a consulté qu’après 20 jours du traumatisme devant la majoration des douleurs. L’examen retrouvait un abdomen sensible, la lipasémie était normale, alors que la tomodensitométrie abdominale montrait un volumineux faux kyste du pancréas (figure 2). Devant l’apparition d’une contracture abdominale le dixième jour de son hospitalisation (à 30 jours du traumatisme abdominal), le patient a été admis au bloc opératoire, l’exploration chirurgicale a montré une rupture du pseudo-kyste dans le péritoine. Il a bénéficié d’un lavage abondant et d’un drainage large ainsi qu’un drainage du faux kyste. L’évolution était favorable avec tarissement progressif du drainage du pseudo-kyste au 7^e^ jour de son hospitalisation.

### Cas numéro 3

Un homme de 50 ans a été victime d’une agression par coup de poing au niveau épigastrique occasionnant des épigastralgies intenses. L’examen trouvait une sensibilité épigastrique. La lipasémie faite après 12 heures était à 4 fois la normale. La TDM montrait une pancréatite stade E sans lésion canalaire pancréatique. Il a été mis sous traitement médical avec une amélioration de sa symptomatologie. L’évolution était marquée par la survenue d’un faux kyste du pancréas après 2 mois. Le patient a été opéré et a bénéficié d’une anastomose kysto-gastrique avec une évolution favorable.

### Cas numéro 4

Un homme de 45 ans a été victime d’un accident de la voie publique avec impact abdominal. L’examen à l’admission trouvait une contracture abdominale généralisée. L’échographie réalisée en urgence a montré un épanchement intra péritonéal de grande abondance et la TDM une pancréatite stade E de Balthazar. Suite à une instabilité hémodynamique, le patient a été admis au bloc opératoire, l’exploration chirurgicale a montré un hémopéritoine de grande abondance avec une contusion pancréatique caudale sans rupture du canal de Wirsung. Il a bénéficié d’une toilette péritonéale avec drainage de la loge pancréatique. L’évolution était favorable.

### Cas numéro 5

**Figure 3:**
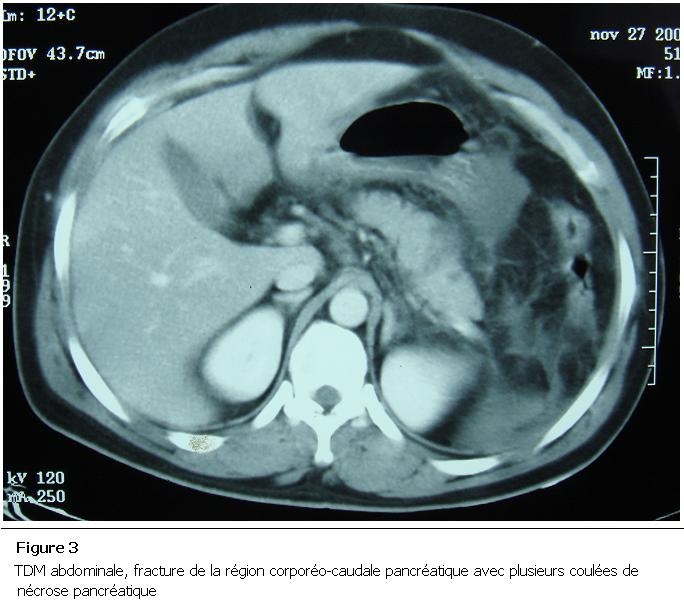
TDM abdominale, fracture de la région corporéo-caudale pancréatique avec plusieurs coulées de nécrose pancréatique

**Figure 4:**
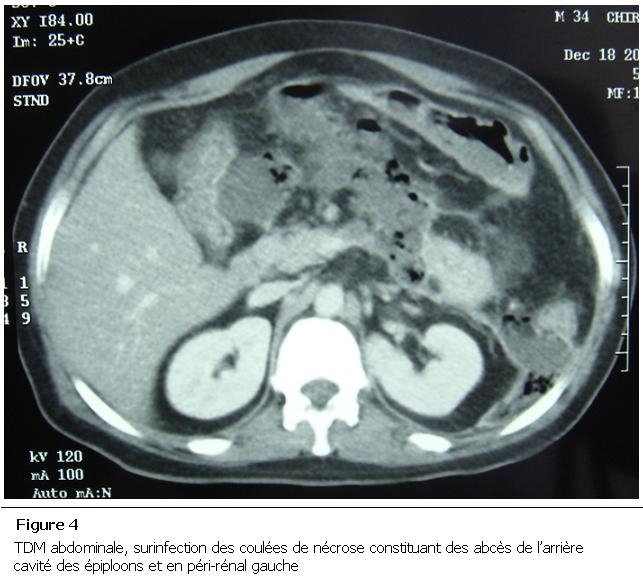
TDM abdominale, surinfection des coulées de nécrose constituant des abcès de l’arrière cavité des épiploons et en péri-rénal gauche

Un homme de 35 ans a été victime d’un accident du travail (réception sur l’abdomen d’une barre métallique tombée de haut) occasionnant des épigastralgies. L’amylasémie était à 1,6 fois la normale et la lipasémie à 12 fois la normale. La TDM a montré une fracture de la région corporéo-caudale pancréatique (Classe II Lucas) avec pancréatite stade E de balthazar (figure 3). Le patient a été traité médicalement. L’évolution était marquée par la surinfection très étendue des coulées de nécrose constatée le 7^e^ jour de son hospitalisation (figure 4). Le patient a été admis au bloc opératoire, a bénéficié d’une nécrosectomie, avec toilette et drainage. Il est décédé 48 heures plus tard des suites d’un choc septique.

## Discussion

La situation profonde du pancréas dans le rétropéritoine explique le faible pourcentage des TIP. Son atteinte relève le plus souvent d’un traumatisme épigastrique violent: accident de la voie publique dans 60 % des cas ou chute de hauteur avec écrasement contre un objet contondant [8]. En raison de son manque de mobilité, le pancréas sera surtout menacé par la lordose du rachis contre lequel il vient s’écraser. Ces lésions peuvent être méconnues et n’être découvertes qu’au stade des complications : pancréatite aiguë, abcès, pseudo-kyste [9]. Chez quatre de nos patients le diagnostic du traumatisme pancréatique a été fait au stade de pancréatite aiguë. Chez le cinquième il a été obtenu au stade de pseudo-kyste.

Les patients souffrant d’TIP sont souvent asymptomatiques les premières heures. Une fois la pancréatite aiguë est installée les douleurs s’aggravent et apparaît la défense abdominale due à l’irritation péritonéale par les coulées de nécrose pancréatique [10]. Quand la lésion pancréatique est peu importante les douleurs peuvent disparaitre rapidement et ne réapparaître qu’avec la survenue d’un pseudo-kyste. C’est le cas d’un de nos patients.

Les examens biologiques utilisés dans un contexte de traumatisme abdominal avec suspicion de lésion pancréatique est le dosage sérique, urinaire ou péritonéal de l’amylase et/ou de la lipase. Ces examens manquent de sensibilité et surtout de spécificité en particulier dans le contexte traumatique (20 à 50 %) [11], mais leur dosage est néanmoins utile en association avec une tomodensitométrie, car une amylase sérique supérieure à 200 UI/L et une lipase sérique supérieure à 1800 UI/L sont en faveur d’une atteinte du canal de Wirsung [12]. La lipasémie a été réalisée chez 4 de nos 5 patients, elle était négative dans 25% des cas.

La tomodensitométrie abdominale (TDM) est l’examen de référence, performant et non invasif, chez le blessé en stabilité hémodynamique présentant un traumatisme de l’abdomen. Cet examen est réalisé de façon optimale en mode spiralé avec injection de produit de contraste. Comme pour la clinique et la biologie, il y a près de 40 % de faux négatifs dans les premières heures qui suivent l’accident [13]. Il faut savoir renouveler la TDM lorsque l’imagerie initiale est atypique. Au stade initial, les images évocatrices d’une lésion pancréatique sont le plus souvent peu spécifiques (épanchement liquidien intra-péritonéal, anomalies du rehaussement de la glande, aspect hétérogène, présence d’une collection dans l’arrière cavité des épiploons). Les signes plus spécifiques sont l’élargissement de la glande, la présence d’une fracture pancréatique hypodense, un aspect d’hématome spontanément hyperdense, mieux individualisé après injection, la présence de liquide entre la veine splénique et la face postérieure du pancréas et l’infiltration de la graisse péri pancréatique et/ou des fascias para-rénaux antérieurs [4, 14]. La preuve d’une atteinte du canal de Wirsung n’est obtenue par TDM que chez la moitié des traumatisés du pancréas [14] mais les progrès récents des scanners multi-barrettes augmentent significativement les performances de la TDM.

Tous nos patients ont eu des TDM. La fracture pancréatique a été mise en évidence dans 2 cas.

La cholangio-pancréatographie rétrograde endoscopique (CPRE) permet de réaliser une cartographie canalaire complète, avec une sensibilité de 100 % pour la détection des anomalies [15]. Elle doit être pratiqué le plus tôt possible, de préférence lors des premières 12 à 24 heures qui suivent le traumatisme [11, 15]. Cependant, elle n’est pas dénuée de risque infectieux sur des tissus pancréatiques contus. La cholangio-pancréatographie par résonance magnétique (CPRM) a une sensibilité de 87 à 100 % et une spécificité de 81 % [16, 17], elle permet de remplacer la CPRE car elle est non invasive. L’apport de l’imagerie par résonance magnétique (IRM) dans l’exploration parenchymateuse ne semble pas supérieur à celui de la TDM [16]. Ces principales limites sont sa disponibilité limitée et la présence chez le patient de matériel d’ostéosynthèse non adapté.

La classification la plus intéressante et la plus utilisée dans les traumatismes pancréatiques est la classification de Lucas [7] (tableau 1) car elle tient compte à la fois de la localisation de la lésion dans le pancréas et de l’existence ou non d’une atteinte canalaire ou duodénale. Elle est assez proche du score de gravité classique rédigé par l’American Association for the Surgery of Trauma (AAST) [18]. Seules les classes I, II et III (TIP) nous intéressent dans cette présentation.

**Tableau 1:** Classification des traumatismes pancréatiques selon Lucas

Classe I	Classe II	Classe III	Classe IVa	Classe IVb
Contusion ou lacération pancréatique avec une atteinte parenchymateuse limitée. Wirsung intact. Pas d’atteinte duodénale associée	Lacération, perforation ou section complète du corps et de la queue. Suspicion de section du canal de Wirsung. Pas d’atteinte duodénale associée	Écrasement, perforation ou section complète de la tête pancréatique. Pas d’atteinte duodénale associée	Atteinte combinée duodéno- pancréatique. Atteinte pancréatique limitée.	Atteinte combinée duodéno-pancréatique. Atteinte pancréatique sévère (rupture du canal de Wirsung).

Kim HS, DK Lee, IW Kim, SK Baik, SO Kwon, JW Park, NC Cho, and BS Rhoe. The role of endoscopic retrograde pancreatography in the treatment of traumatic pancreatic duct injury. Gastrointest Endosc. 2001; 54(1):49–55. This article on PubMed

L’attitude thérapeutique devant les TIP dépendra du stade lésionnel. L’option non opératoire doit être réservée aux patients présentant un traumatisme abdominal fermé chez qui il existe une convergence d’arguments cliniques et para-cliniques en faveur d’une lésion pancréatique isolée sans rupture du canal de Wirsung (classe I de Lucas). Le traitement médical d’une contusion pancréatique est inspiré de celui de la pancréatite aiguë biliaire qui a fait l’objet d’une conférence de consensus [19] : sonde naso-gastrique seulement en cas de vomissements importants, apport hydro-électrolytique adéquat, antalgiques (essentiellement paracétamol et morphiniques) adaptés à l’évaluation visuelle analogique. L’antibiothérapie préventive est discutable dans ce contexte et n’est pas a priori proposée.

Lors d’une rupture du canal de Wirsung (classe II et III) objectivée par la CPRM et/ou la CPRE préopératoire, la mise en place d’une prothèse endo-canalaire a donné d’excellents résultats, que la lésion soit céphalique ou corporéo-caudale, chez l’enfant comme chez l’adulte dans des centres expérimentés [14, 20]. Lorsque ce geste n’est pas réalisable, il existe une alternative thérapeutique : dans un premier temps, un traitement médical et une surveillance en milieu chirurgical, suivie d’un éventuel geste de dérivation interne si un pseudo-kyste survient (dérivation kysto-gastrique ou kysto-jéjunale), ce qui serait le cas de 80 % des blessés présentant une atteinte canalaire d’autant plus chez l’enfant [21]. Cependant, chez l’adulte, le traitement chirurgical reste indiqué devant l’atteinte du canal de Wirsung et s’il y a un doute sur une lésion associée.

Un de nos jeunes patients (cas n°1, 17 ans) n’a pas eu de complications malgré la présence d’une fracture isthmique sur le scanner. Cependant, chez le patient n°5 (35 ans), la fracture pancréatique s’est rapidement compliquée d’une pancréatite suivie de la constitution rapide d’un abcès.

Dans les classes II de Lucas, la chirurgie consistera en une pancréatectomie gauche avec préservation de la rate plutôt qu’une spléno-pancréatectomie gauche car cette dernière expose au risque infectieux [22]. Dans les classes III, la suture du pancréas céphalique accompagné d’une anastomose du pancréas gauche sur une anse en Y ou sur l’estomac est une intervention possible [23]. C’est une alternative à la duodéno pancréatectomie céphalique qui est indiquée en cas de contusion avec destruction de la tête du pancréas ne permettant pas de la préserver. Le simple drainage de contact sera réalisé si l’état hémodynamique du patient ne permet pas de prolonger l’intervention.

La mortalité globale chez les patients atteints de traumatisme abdominaux avec atteinte pancréatique varie de 5 à 30 % selon les séries [2, 3, 8, 13, 24], cependant, elle n’est directement imputé au pancréas que dans 5 à 10 % de ces décès [2, 3, 24].

## Conclusion

La rareté des TIP et leur symptomatologie clinique atypique au stade de début rend leur diagnostic relativement difficile d’autant plus que la biologie n’est pas spécifique. Ceci doit nous pousser à réaliser des TDM devant tout traumatisme épigastrique même s’il n’est pas violent. La mise en place de prothèse pancréatique constitue une évolution remarquable dans le traitement actuel des TIP. La chirurgie de résection pancréatique réalisée devant une rupture canalaire mais améliore le pronostic des TIP malgré une morbidité non négligeable.

## Conflits d’intérêts

Les auteurs ne déclarent aucun conflit d’intérêts.

## Contribution des auteurs

**HEB**, **AO**, **KIM**, **KM**, **AL** et **KAT** ont opéré les patients; **AA** et **AB** ont contribué à la recherche bibliographique. Tous les auteurs ont lu et approuvé la version finale du manuscrit.
